# A Study on the Effect of Mental Practice Using Motor Evoked Potential-Based Neurofeedback

**DOI:** 10.3389/fnhum.2021.637401

**Published:** 2021-02-12

**Authors:** Daiki Matsuda, Takefumi Moriuchi, Yuta Ikio, Wataru Mitsunaga, Kengo Fujiwara, Moemi Matsuo, Jiro Nakamura, Tomotaka Suzuki, Kenichi Sugawara, Toshio Higashi

**Affiliations:** ^1^Graduate School of Biomedical Sciences, Nagasaki University, Nagasaki, Japan; ^2^Department of Occupational Therapy, Nagasaki Memorial Hospital, Nagasaki, Japan; ^3^Faculty of Health and Social Work, Division of Physical Therapy, Kanagawa University of Human Services, Yokosuka, Japan

**Keywords:** mental practice, motor evoked potential, neurofeedback, transcranial magnetic stimulation, motor imagery training

## Abstract

This study aimed to investigate whether the effect of mental practice (motor imagery training) can be enhanced by providing neurofeedback based on transcranial magnetic stimulation (TMS)-induced motor evoked potentials (MEP). Twenty-four healthy, right-handed subjects were enrolled in this study. The subjects were randomly allocated into two groups: a group that was given correct TMS feedback (Real-FB group) and a group that was given randomized false TMS feedback (Sham-FB group). The subjects imagined pushing the switch with just timing, when the target circle overlapped a cross at the center of the computer monitor. In the Real-FB group, feedback was provided to the subjects based on the MEP amplitude measured in the trial immediately preceding motor imagery. In contrast, the subjects of the Sham-FB group were provided with a feedback value that was independent of the MEP amplitude. TMS was applied when the target, moving from right to left, overlapped the cross at the center of the screen, and the MEP amplitude was measured. The MEP was recorded in the right first dorsal interosseous muscle. We evaluated the pre-mental practice and post-mental practice motor performance in both groups. As a result, a significant difference was observed in the percentage change of error values between the Real-FB group and the Sham-FB group. Furthermore, the MEP was significantly different between the groups in the 4th and 5th sets. Therefore, it was suggested that TMS-induced MEP-based neurofeedback might enhance the effect of mental practice.

## Introduction

With the progress in brain imaging technology in recent years, mechanisms in the brain that have been treated as black boxes are gradually being clarified. In particular, even when the brain is damaged, such as in cerebrovascular disorders, it has been proven that plastic brain changes, as a consequence of training, do occur. In recent years, attention has been focused on rehabilitation based on knowledge of the mechanisms in the brain. Under such circumstances, mental practice (i.e., motor imagery training) is one of the means of rehabilitation acting in complement to movement therapy; mental practice is a method of repeatedly reproducing motor imagery.

Motor imagery is the execution of a mental action in a state where there is no clear movement or muscle activation (Mizuguchi et al., 2014). It has been reported that in motor imagery, activation in the brain is recognized to be almost similar to the actual action, without the accompanying movement. Specifically, studies using positron emission tomography (PET) and magnetic resonance imaging (fMRI) have reported that the premotor area, supplementary motor area, cingulate, and parietal cortex are activated during these mental exercises (Porro et al., 1996, 2000; Deiber et al., 1998; Lotze et al., 1999; Gerardin et al., 2000; Ehrsson et al., 2003; Hanakawa et al., 2003; Jackson et al., 2003; Kuhtz-Buschbeck et al., 2003; Dechent et al., 2004; Meister et al., 2004). In addition, activation of the primary motor cortex (M1) has been reported by studies using electroencephalography (EEG) and transcranial magnetic stimulation (TMS) (Pfurtscheller and Neuper, 1997; Hollinger et al., 1999; Caldara et al., 2004; Mattia et al., 2004). Mental practice does not require special machines or devices, and subjects can easily work on it without any time or space restrictions. Furthermore, since it can be carried out without actual movement, it can be applied to patients who do not have the capacity to perform voluntary exercise and minimize dangers such as risk of falling (Dietrich, 2008). In fact, there is also a report, using randomized controlled trials, that demonstrates the effect of mental practice on the improvement of upper limb paralysis in stroke patients (Page et al., 2009; Nilsen et al., 2012; Park and Lee, 2015). Furthermore, a systemic review showed that mental practice is an effective intervention for upper limb dysfunction in stroke patients (Langhorne et al., 2009).

However, although evidence has been shown for the effect of mental practice, it is not widely used in clinical settings. The reason for this is that since motor imagery is processed in the brain, it is difficult to objectively evaluate how clearly the subject undertakes motor imagery as a task, from the viewpoint of the therapist. Therefore, it is difficult to give accurate feedback during practice. To address this problem, attempts to evaluate brain activity in motor imagery using brain imaging technology and provide feedback to the subject in real-time, have been reported (Broetz et al., 2010; Ang et al., 2011; Pichiorri et al., 2015; Mehler et al., 2019). For example, a previous study measured the change in cerebral blood flow rate in a motor-related region (right dorsal region of the premotor cortex), as a blood oxygenation level-dependent signal using fMRI. Through feedback to participants, it was possible to perform mental practice while maintaining increased motor imagery ability; as a result, the study reported an improvement in performance (Hui et al., 2014). In addition, oxygenated hemoglobin signals in the premotor area, on the opposite side, were recorded with motor imagery using near-infrared spectroscopy (NIRS); we performed the measurements by dividing participants in to the real-FB group (feedback of the correct amount of cerebral blood flow change to the subject) and the sham-feedback group (feedback of a false cerebral blood flow change). The real-FB group showed that the self-assessment scale scores for kinesthetic motor imagery were higher than those of the Sham-feedback group (Mihara et al., 2012). However, the abovementioned neuroimaging equipment is expensive, highly restrictive, and possibly difficult to widely use in the clinic. Conversely, TMS is a method that can evaluate brain activity during motor imagery, like to be using fMRI and NIRS. The motor evoked potential (MEP) is widely used as an evaluation of M1 excitability, and the MEP amplitude (peak-to-peak) has been reported to be significantly higher during motor imagery than in control conditions in several previous studies (Fadiga et al., 1998; Facchini et al., 2002; Munzert et al., 2009).

It has been reported that a greater MEP amplitude is associated with greater motor imagery (Williams et al., 2012) and more vivid kinesthetic motor imagery (Ohno et al., 2011; Ikeda et al., 2012; Moriuchi et al., 2020). Based on the above, it is possible that results similar to the results of feedback performed by fMRI and NIRS can be obtained even when feedback using TMS is performed. Compared to fMRI and NIRS, TMS equipment is relatively inexpensive and easy to move, so there are few restrictions; as such, we thought that it would be useful for increasing opportunities for neurofeedback in clinical situations.

Therefore, the purpose of this study was to verify whether M1 excitability can be promoted by feedback to the subject with the MEP amplitude (peak-to-peak) induced by TMS as an index.

## Materials and Methods

### Subjects

A power analysis using G-power revealed a requisite sample size of 22 (with an effect size of 0.4 and significance level of *p* < 0.05, power of 0.8). A total of 24 healthy subjects (14 men and 10 women; mean age, 22.4 ± 3.4 years) were enrolled in the study. All participants provided written informed consent, and all were right-handed (as indicated by self-report). None of the subjects reported neurological impairment or contraindications to TMS. The study was approved by the local ethics committee of Nagasaki University Graduate School of Biomedical Sciences. All experimental procedures were conducted in accordance with the Declaration of Helsinki (World Medical Association, 2013).

To reveal the effect of TMS feedback, all participants were randomly allocated to either the real-feedback group (Real-FB) (*n* = 12; a group given a right MEP amplitude) or the sham-feedback group (Sham-FB) (*n* = 12; a group given a non-related value of MEP amplitude).

### Experimental Set-Up

**Figure 1:**
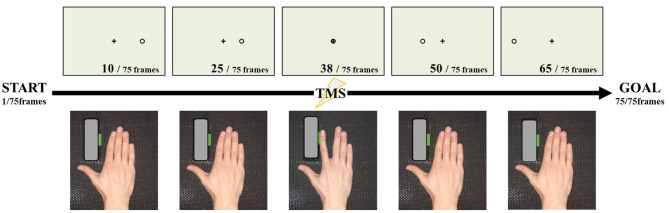
Experimental set-up and task. Seventy-five images were continuously reproduced, and magnetic stimulation was applied at the time of the 38th frame at which the cursor overlapped with the target point. At the time when the cursor overlapped with the target point, we instructed the participant to press the button by index finger abduction.

Subjects were seated on a reclining chair 80 cm away from a computer monitor (19-inch, resolution 1,024 × 768 pixels, refresh frequency 60 Hz) and were instructed to keep both hands in a pronated position on a horizontal board attached to the chair's armrest. They were instructed to keep the right forearm as still and relaxed as possible while paying attention to the visual stimuli presented on the monitor. The position of the right index finger was adjusted, as shown in Figure 1, so that the switch could be pressed by the abduction movement.

### Experimental Task

The motor imagery task was conducted as shown in Figure 1. The timing of the TMS trigger needs to stimulate at the same point as the action, during motor imagery. Therefore, in the present study, we adopted the coincidence timing task, which involved pressing the button through index finger abduction, coinciding with the arrival of a cursor, running on a straight forward line from the start point. In the performance evaluation of this experiment, the abduction switch was pressed with the index finger touching the button, such that the abduction angle was very small. The starting point was to the right of the monitor and the target point was in the middle of the screen.

The experimental video was made from 75 individual JPEG files, from the starting point to the end point, and shown in succession to obtain the animation effect, which was presented at a speed of 33.3 ms/frame. The timing of the coincidence with the arrival of the cursor to the target point was the 38th file. Based on the above, the circle reached the target in 1.27 s, and the distance from the start position to the target was 12.5 cm on the monitor. One set (20 trials) was used for performance evaluation.

An experimental movie was played where a black cross in the center of a white screen was presented. After the warning signal (beeping sound), the cursor ran on a straight line at a constant speed from the starting point, to the target point. Subjects were required to pay attention to the movement of the cursor on the monitor and to press the button with index finger abduction, when the cursor arrived at the target point. The experimental program used in the present study was a custom-made program by LabVIEW systems (LabVIEW, National Instruments, USA).

### Mental Practice

Mental practice with motor imagery was conducted for five sets with 20 trials per set. Therefore, the total number of trials was 100. Subjects were instructed to kinesthetically imagine the coincidence timing task as if they were actually performing the movement, and subsequently recall the sense of the fingertip, muscle strength, and the sound when they pressed the button.

### TMS Feedback

**Figure 2:**
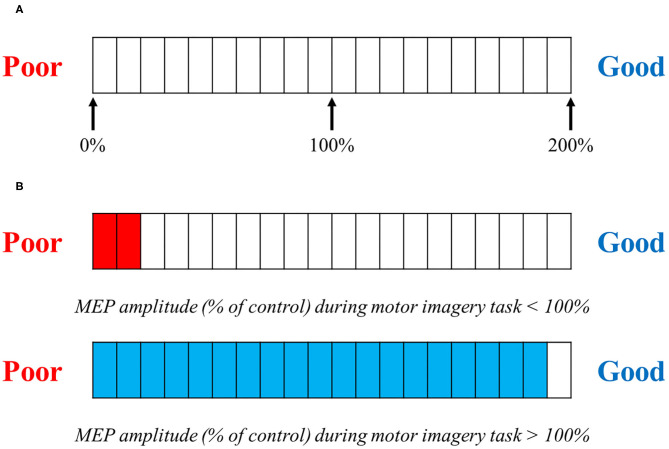
TMS feedback. **(A)** Motor evoked potential (MEP) amplitudes compared with baseline were feedback visually on each trial. **(A)** shows the level meter displayed to the subject when providing feedback. This is represented by a graduation that ranges from 0 to 200%, in 10% increments. The value in the motor imagery task, compared with the MEP at rest, is displayed. **(B)** If the MEP amplitude size during the motor imagery task is <100% compared with that in the control (at rest) MEP amplitude size, the level meter is displayed in red; if the MEP amplitude is over 100%, the level meter is displayed in blue.

We used MEP induced by TMS during motor imagery for neurofeedback. The TMS trigger was set at the timing of the arrival of a cursor to the target point. Subjects obtained feedback of the obtained MEP amplitude values. Figure 2A shows the monitor of the neuro-feedback system used in the present study. We set the level meter, at the bottom of the monitor, which could reflect the corticospinal excitability during motor imagery. A value of 100% indicated corticospinal excitability in the resting condition. This level meter was represented by a scale from 0 to 200%, where every 10% indicated the relative change in corticospinal excitability during motor imagery (Figure 2B). A previous study reported that the difference in vividness for motor imagery affected corticospinal excitability (Moriuchi et al., 2020). Therefore, if subjects can imagine something vividly, the value of the level meter reaches over 100%. On the other hand, if subjects cannot imagine vividly, the value of the level meter will be equal to or <100%.

Corticospinal excitability was assessed in the program and immediately displayed on the level meter. For the Real-FB group, the level meter provided the real value of each trial. However, for the Sham-FB group, the level meter randomly provided a non-related value of the actual corticospinal excitability. The bar displayed by the program is set to vary randomly between 0 and 200%. Therefore, it is considered that it was displayed nearly evenly for the Sham-FB group. The subjects were provided with sufficient prior explanation by the experimenter about the mechanism of the level meter used for feedback. In addition, we explained that the closer to Good, the better the motor imagery, and the closer to Poor, the worse the motor imagery.

### TMS and MEP Recording

Surface EMG signals were amplified and filtered at a bandwidth of 5–3,000 Hz using a digital signal processor (Neuropack Sigma MEB-5504, Nihon Kohden; Tokyo, Japan). Analog outputs from a single processor were digitized at a sampling rate of 2,000 Hz and saved to a computer for offline analysis using an A/D converter (PowerLab16/30, AD Instruments, Sydney, Australia).

At the beginning of the experiment, we identified the optimal TMS coil position for evoking MEPs in the right FDI (the hotspot). TMS was delivered to the left M1 hotspot, marked with a pen on a swimming cap covering the scalp of each subject. TMS employed a 70 mm figure-eight coil connected to a magnetic stimulator (Magstim 200, Magstim, UK). The coil was placed tangentially to the scalp with its handle pointing backward and rotated ~45° away from the mid-sagittal line. Care was taken to maintain the same coil position relative to the scalp throughout the experiment. The resting motor threshold (MT) was defined as the lowest stimulus intensity that evoked a MEP of at least 50 μV in amplitude, in the right FDI, in five out of 10 trials. The test stimulus intensity was set at 110–130% of the resting MT. The mean amplitude of the control MEP for the FDI was 0.5~1.0 mV. Throughout the experiments, subjects were instructed to avoid inadvertent movements that could give rise to background EMG activity. For each muscle in each trial, the 20 ms period preceding TMS triggering was checked for background EMG activity.

### Evaluation

An assessment of performance and vividness for motor imagery was conducted before and after the intervention of mental practice using TMS feedback. Performance assessment was based on how far the target point and the cursor deviated, when the subjects pressed the switch button in the coincidence timing task. Subjects were given 20 trials and we evaluated how many images were displaced with respect to where the target point and the cursor coincided. Calculation of the number of errors was incorporated into the program and could be performed automatically. The maximum value of the error was 37, which was calculated from the target point.

To rate the vividness of the subjects' motor imagery, the subjects were asked to complete a self-evaluation test on a visual analog scale (VAS). Subjects marked a location on a 100-mm horizontal line, the two ends of which were labeled “0 = None at all” and “100 = Very vivid image,” according to the vividness of the imagery they imagined (Martin and Ulrike, 2006; Ikeda et al., 2012).

### Experimental Procedure

We conducted 20 trials of performance evaluation of actual movement in both groups (pre-evaluation). We then measured the MEP amplitude at rest. Subsequently, motor imagery training of pressing the switch, was carried out to ensure vividness of the motor imagery of the subject, and the VAS was used to evaluate the vividness of the subjective motor imagery (pre-evaluation). We then performed a motor imagery task as a mental practice using TMS feedback across five sets (total 100 trials), with 20 trials being one set. After the experimental task, both groups were again evaluated by VAS (post-evaluation), and the performance evaluation of actual movement was conducted for 20 trials (post-evaluation). The two groups were compared and analyzed.

### Data and Statistical Analysis

We compared performance improvement (percentage change of error values), MEP, and changing vividness of motor imagery (percentage change of VAS scores) between the Real-FB group and Sham-FB group. The error value and VAS score were calculated using the following equation: [(the error value of 20 trial or VAS score of the post-test—the error value of 20 trial or VAS score of the pre-test)/the error value of 20 trial or VAS score of the pre-test × 100+100]. An independent *t*-test was used to examine group differences in performance improvement. Furthermore, if a background EMG was found, the data of the trial were rejected. The MEP amplitude (peak-to-peak) was measured in every trial. The data were analyzed statistically using two-way analysis of variance (ANOVA), with the factors “group” (Real-FB vs. Sham-FB), and “trial sets” (rest, 1st−5th sets). The background EMG activities (with each TMS trial data represented as the root-mean-square (RMS) amplitude of the 20 ms prior to the TMS trigger) of right FDI muscles were analyzed using two-way repeated-measure ANOVA, with the factors “group” (Real-FB vs. Sham-FB), and “trial sets” (rest, 1st−5th sets). When a main or interaction effect was found in “trial sets,” a *post-hoc* analysis was conducted using Dunnett's test. On the other hand, if the main or interaction effects was found in “group,” an independent *t*-test was performed to examine group differences for each set.

In all analyses, a *p*-value of < 0.05 was considered statistically significant. All analyses were performed using statistical analysis software (SPSS version 22.0, IBM, USA).

## Results

### Change in Motor Performance

First, a two-way ANOVA was performed in a total of 20 trials using the error value for each trial. The results of the two-way ANOVA for “group” (Real-FB vs. Sham-FB) and “evaluation point” (pre-evaluation vs. post-evaluation) showed that there were significant main effects for “group” and “evaluation point” and a significant interaction. A Box's M test confirmed that p = 0.556; the observed covariance matrix of the dependent variable was equal between the two groups.

**Figure 3:**
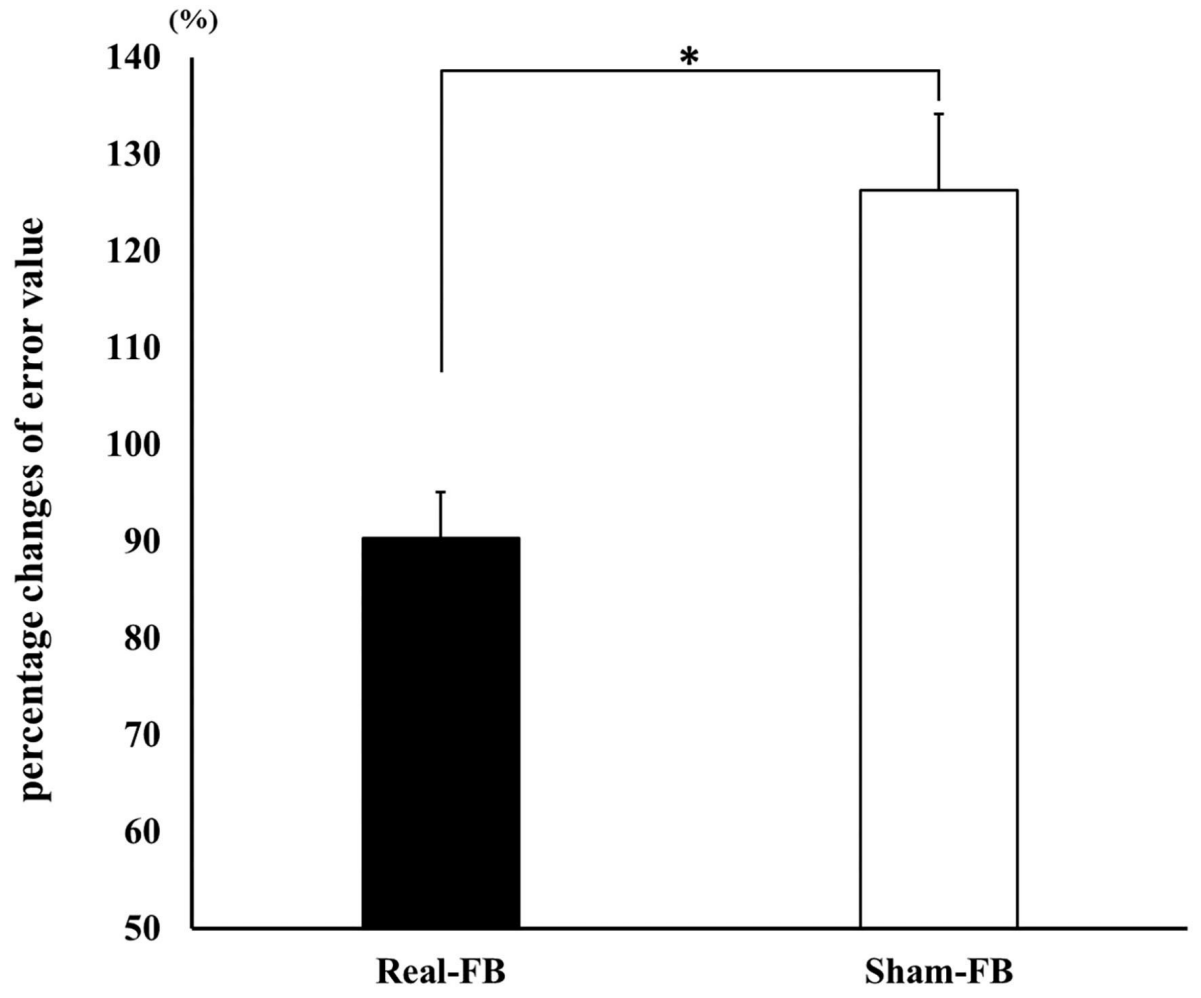
Change in motor performance. Percentage change in the error values (mean ± SE) in each feedback group. *p < 0.05.

Figure 3 shows the motor performance (± standard error) change in both groups. A significant difference was observed in the percentage change of the error values between the Real-FB group and the Sham-FB group. The motor performance uses the errors in 20 trials, thus, a lower value means a better performance. The percentage change in error values was 100% or less in the Real-FB group and 100% or more in the Sham-FB group. In other words, this shows that the timing error decreased in the Real-FB group and increased in the Sham-FB group. Table 1 shows the error value and percentage change of error values, in each group.

**Table 1:** Error value and percentage change of error values for each group.

		**Pre**	**Post**
Real-FB group	Error value	42.3 ± 15.9	38.9 ± 16.9
	Percentage changes of error value	90.4 ± 16.6	
Sham-FB group	Error value	35.2 ± 11.8	43.8 ± 15.2
	Percentage changes of error value	126.3 ± 27.6	

### Change in MEP Amplitude During Mental Practice

**Figure 4:**
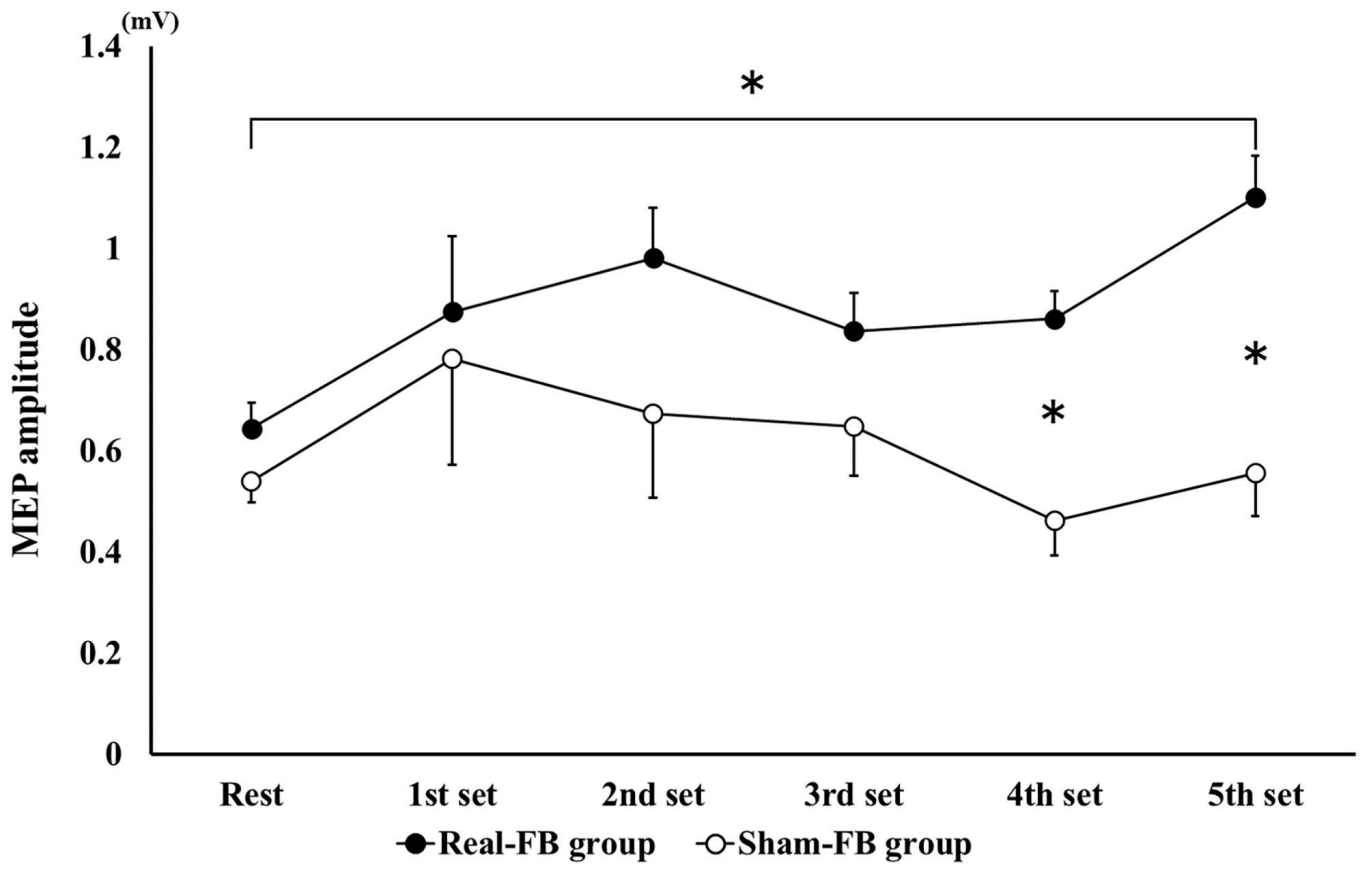
MEP amplitude change in each set during the motor imagery task. Changes in MEP amplitude (mean ± SE) for each feedback group. *p < 0.05.

**Figure 5:**
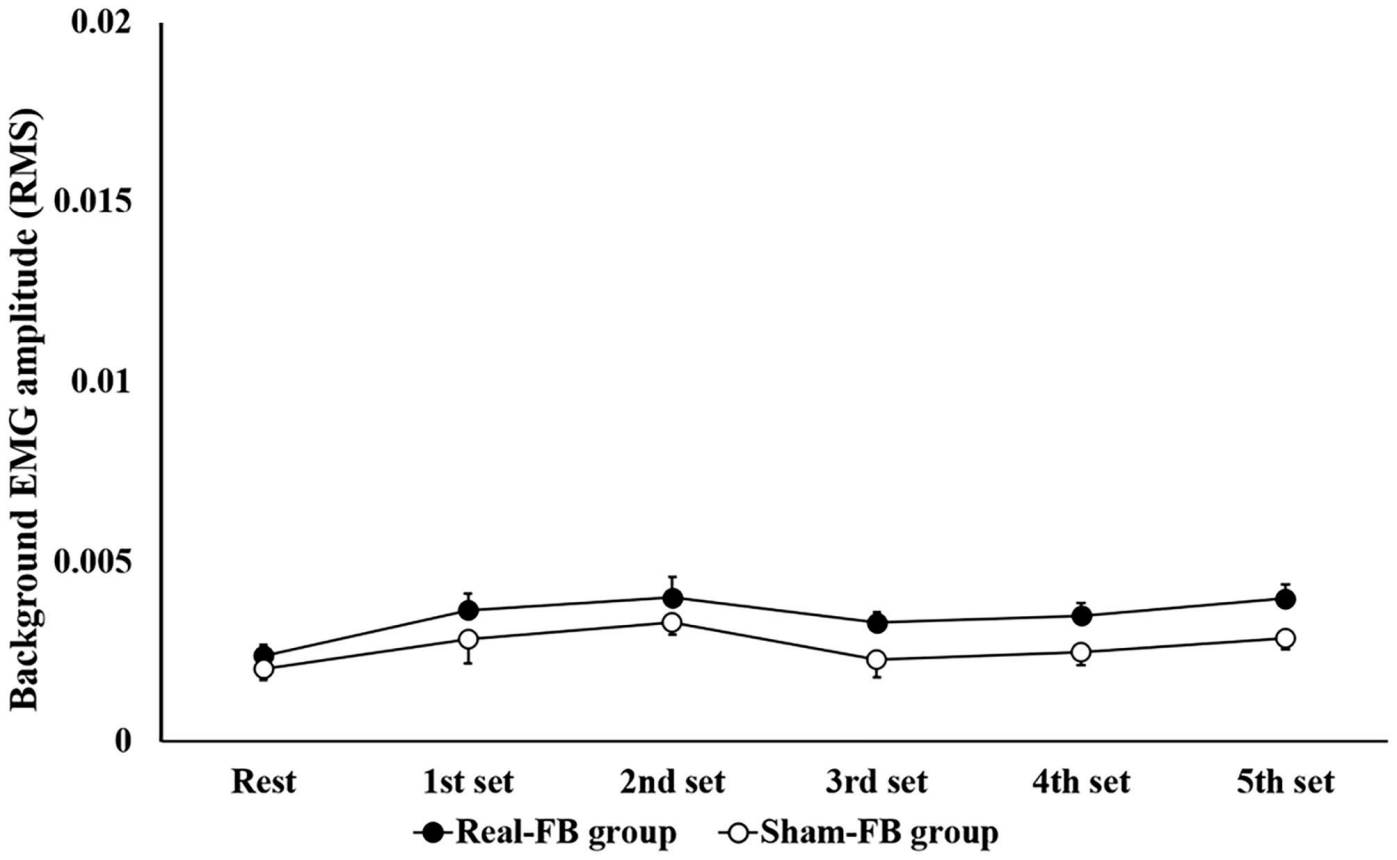
Background EMG amplitude in each set during the motor imagery task. Changes in root mean square of the background EMG amplitude (mean ± SE) for each feedback group.

Subjects in the Real-FB group had a >100% MEP compared to rest MEP, with the 1st set corresponding to 116/240 trials, 2nd set corresponding to 142/240 trials, 3rd set corresponding to 125/240 trials, 4th set corresponding to 136/240 trials, and 5th set corresponding to 164/240 trials. Among individuals, individuals achieved more than 100% of MEP compared to rest MEP in about 30/100–80/100 trials. Figure 4 shows the change in the mean MEP amplitude (± standard error) in both groups. The two-way ANOVA showed a significant main effect for “trial sets” and “group.” Dunnett's *post-hoc* test revealed that there was a significant difference between the rest set and the 5th set of MEPs. Moreover, an unpaired *t*-test between groups of Real-FB group and Sham-FB group found no significant difference in the MEP amplitude at rest, but a significant difference was observed in MEP amplitudes in the 4th and 5th sets (*p* < 0.01). A Box's M test confirmed that *p* = 0.458; the observed covariance matrix of the dependent variable was equal between the two groups. Figure 5 shows the changes in the RMS background EMG amplitude (in both groups. The two-way ANOVA showed no significant main effect and interaction for “trial sets” and “group” of the background EMG.

### Change in the Vividness (VAS) of Subjective Motor Imagery

**Figure 6:**
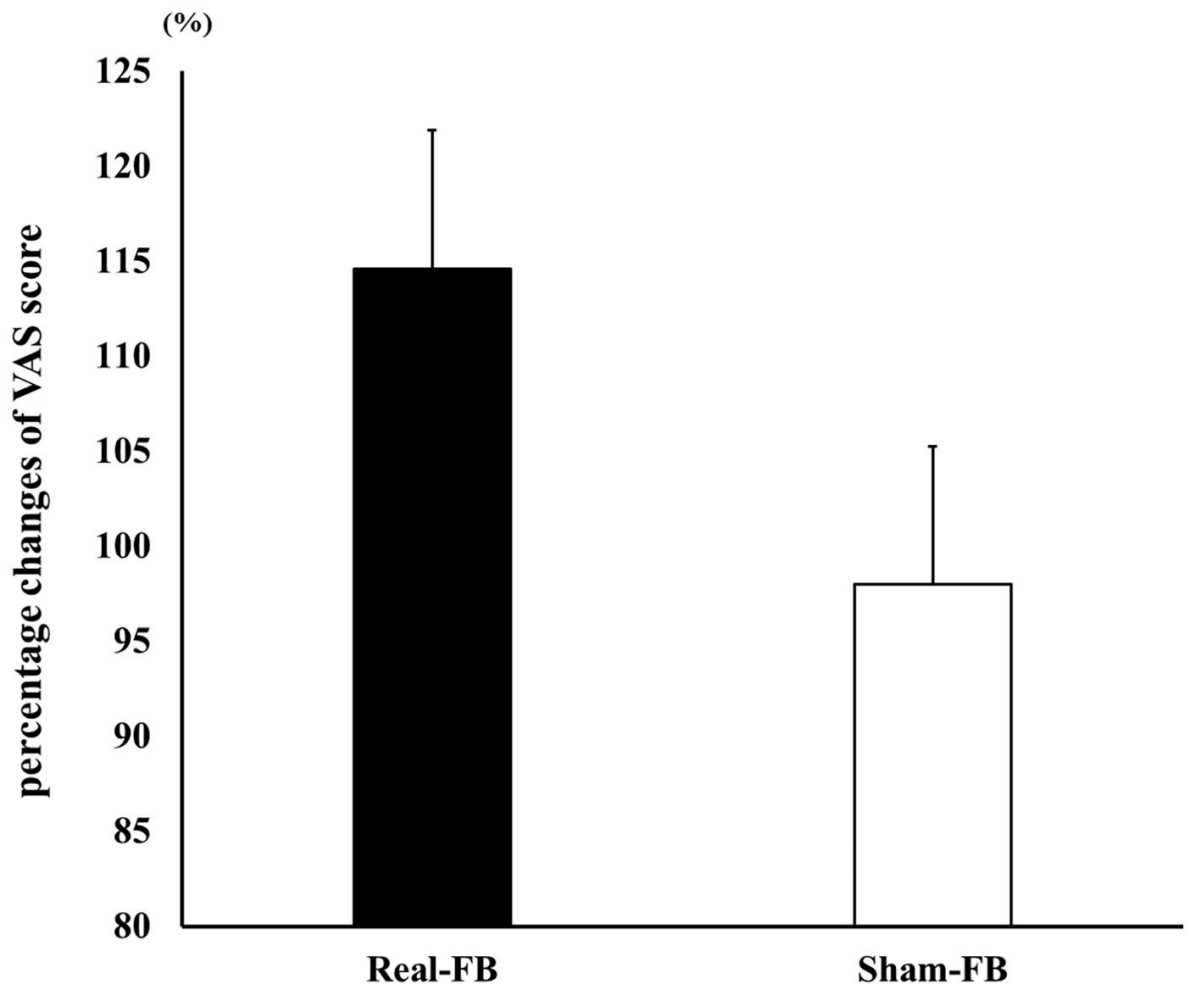
Percentage change in VAS score. Percentage change in the VAS score (mean ± SE) in each feedback group.

Figure 6 shows the change in VAS (± standard error) in both groups. The results of the two-way ANOVA showed no significant interaction between groups and trials. A Box's M test confirmed that *p* = 0.736; the observed covariance matrix of the dependent variable was equal between the two groups. Furthermore, there was no significant difference in the percentage change in VAS scores; however, there was a tendency for improvement in the Real-FB group's VAS score in the “evaluation point” (*p* = 0.059). Table 2 shows the VAS score and percentage change of VAS values of each groups.

**Table 2:** VAS score and percentage change of VAS values of each groups.

		**Pre**	**Post**
Real-FB group	Score (mm)	70.2 ± 12.3	80.4 ± 17.8
	Percentage changes of VAS value (%)	114.6 ± 25.2	
Sham-FB group	Score (mm)	71.3 ± 11.0	70.0 ± 14.6
	Percentage changes of VAS value (%)	98.0 ± 25.2	

## Discussion

The purpose of this study was to clarify whether the effect of mental practice is enhanced when providing neurofeedback based on TMS-induced MEP as an index. In order to clarify the effect of TMS feedback, we divided the subjects into two groups; the Real-FB group which received a feedback MEP amplitude as an index and the Sham-FB group which received feedback values not related to MEP amplitude. The two groups were then compared, and a significant difference was observed in the percentage change of error values between the Real-FB group and the Sham-FB group. Furthermore, the MEP was significantly different between the groups in the 4th and 5th sets. On the other hand, there were no significant differences found in VAS scores. However, there was an improvement tendency between pre- and post-mental practice in the Real-FB group.

### Change in Motor Performance

In this study, we used the coincidence timing task, which involved pressing a button by index finger abduction. As a result, there were significant differences in the percentage change of error values. Shenghong et al. (2020) reported that the effect of neurofeedback training during simple motor imagery was only significant in the real-feedback group but not in the sham group. Moreover, the previous study used real-time fMRI neurofeedback-guided motor imagery based on contralateral M1 hand area blood oxygen level dependent (BOLD) signals in healthy subjects and found positive correlations between contralateral M1 activation and performance changes in the motor imagery task as an isometric force precision grip task (Blefari et al., 2015). In other words, subjects with stronger contralateral M1 activation during motor imagery may benefit more from mental practice. These findings support the result of the present study that performance was improved by using MEP amplitude and reflected the excitability of the corticospinal tract including the M1. However, we examined the short-term performance change in only 100 trials. In previous studies on the effectiveness of mental practice, researchers examined the effect of mental practice on short-term performance change with the hand sequence task, most of which examined the effects within weeks to months (Yasushi et al., 2010; Frenkel et al., 2014; Avanzino et al., 2015; Di Rienzo et al., 2015). Therefore, we think it is necessary to verify the long-term intervention effects of feedback using TMS.

### Change in MEP Amplitude During Mental Practice

In this study, there were significant main effects in “trial sets” and “group”; there was a significant difference between rest and the 5th set of MEPs in the Real-FB group. Moreover, a significant difference was observed in MEP amplitudes at the 4th and 5th sets (*p* < 0.01).

Therefore, it was suggested that M1 excitability during mental practice is kept higher in the Real-FB group than in the Sham-FB group. Such a result may have been obtained potentially because in the Real-FB group, true feedback of M1 excitability in motor imagery is accurate feedback. As in the present study, Mihara et al. used NIRS to assess brain activity feedback near the contralateral premotor cortex during motor imagery in a real-FB group. They reported that cerebral cortex activity was increased centered on the contralateral premotor cortex but decreased near the dorsolateral parietal association cortex. Furthermore, operant conditioning, which provides the size of MEP feedback, shows that participants can self-modulate their own brain state (for example, by providing feedback according to the MEP size, MEP could be increased for UP training sessions and conversely decreased for DOWN training sessions) (Kathy et al., 2018). In this study, we thought that the MEP during MI in the Real-FB group could be maintained at a high value by feedback of the MEP as a bar.

In contrast, in the Sham-FB group, it is thought that confusion occurred because it was difficult to judge the correctness of the motor imagery itself through introspection of the motor imagery and the gap of the feedback result. Mihara et al. (2012) reported that under sham conditions, subjects could feel uncertain and lose confidence in kinesthetic imagery with incorrect feedback, which could mislead the subjects. In this study, it is possible that the feedback was not stable, such as in the form of Good or Poor feedback, even though the motor imagery was performed in the Sham-FB group. In addition, there is a possibility that the timing of the motor imagery may be questionable, or anxiety may have been caused whereby the motor imagery may not have been created; it is thought that MEP decreased due to confusion.

Based on the above observations, it was suggested that the MEP amplitude-based feedback used in this study could maintain high MEP during MI and may enhance the effect of mental practice.

### Change in Vividness of Subjective Motor Imagery

In this study, subjective motor imagery vividness was evaluated using the VAS. As a result, there was no significant difference in the percentage change in VAS score. However, there was an improvement tendency between pre- and post-mental practice in the Real-FB group.

A feedback study using NIRS reported by Mihara et al. (2012) showed that subjective motor imagery vividness was significantly higher in the Real-FB group than in the Sham-FB group. Unfortunately, although there was a tendency, we could not show a statistically significant difference in this study. However, as mentioned above, there was a significant difference between the two groups in the MEP amplitude in the motor imagery task. In the Real-FB group, a high MEP was maintained in the 5th set, and it was reported that MEP is highly related to the vividness of subjective motor imagery (Moriuchi et al., 2020). Therefore, we considered that a tendency to improve in the Real-FB group VAS score was observed. Moving forward, we think that it is necessary to examine long-term intervention effects as well as performance changes.

### Limitations

Referring to the study of Mihara et al. (2012), we compared two groups, the Real-FB group that received feedback on the MEP amplitude as an index and the Sham-FB group that received feedback values that were not related to the MEP amplitude. However, this study design does not show the difference in effect from the case where feedback is not given. Searching for studies that verified the effects of other neurofeedback, we found that studies that set groups that do not provide feedback, as a control group (deCharms et al., 2005), or those that adopted a region other than the target region, or a signal from a third party for feedback of the sham-FB group (Subramanian et al., 2011; Sitaram et al., 2012; Young et al., 2014), have also been reported. Taking these reports into consideration, setting a group to not receive feedback as a control group, could have shown an effect of feedback using TMS on mental practice. In addition, as mentioned above, this study merely examined the effect of short-term mental practice on healthy subjects. In the future, to clarify effective methods of mental practice in rehabilitation, it is necessary to investigate the change in performance by long-term intervention settings and examinations for stroke patients, for example.

### Clinical Implications

Mental practice using fMRI and NIRS is considered to be difficult to perform in clinical terms from the viewpoint of constraint and mobility. We hypothesized that performing mental practice using TMS would be more feasible to use in clinical settings. As a result of this study, a significant difference was observed in the percentage change of error values between the Real-FB group and the Sham-FB group. Furthermore, there was a significant difference between the rest set and the 5th set of MEPs. Recent studies on mental practice using neurofeedback have reported that accurate feedback of brain status can maintain high vividness of motor imagery and performance improvement was observed. Similar to these findings, in this study, maintenance of increased MEP amplitude was observed in the Real-FB group compared with the resting amplitude. It seems that mental practice could be performed while maintaining the vividness of the high motor imagery corrected by feedback of M1 excitability; mental practice using TMS seems to be effective. In this study, we examined only the short-term effects; however, we will consider that a similar performance improvement as in fMRI and NIRS studies would be observed by verifying the long-term effects.

In addition, changes in M1 excitability are also being evaluated in various TMS-based studies at all stages from the acute phase to the chronic stage, after stroke (Cicinelli and Traversa, 1997; Escudero et al., 1998; Delvaux et al., 2003; van Kuijk et al., 2008; McDonnell and Stinear, 2017; Mooney et al., 2020). In other words, although there are restrictions such as consciousness level, since the MEP amplitude can be derived at any stage after stroke, we consider that neurofeedback using MEP amplitude can be used at any stage of stroke. Regarding the use of this method in a clinical setting, we couldn't verify the effect of giving feedback in patients only with the results of this study, so we considered that this method is inadequate for use in a clinical setting. However, it was suggested that giving real feedback could maintain the increased MEP amplitude, compared with the resting amplitude in healthy subjects; therefore, with mental practice while giving real feedback using TMS, mental practice can be carried out while maintaining higher motor imagery vividness, which may be recognized as an improvement in function.

## Conclusion

Feedback using MEP amplitude, induced by TMS as an index, suggested the possibility of enhancing the effect of mental practice because enhanced M1 excitability during mental practice was observed. Thus, in future studies, it is necessary to verify the comparison with the control group and long-term effects of intervention.

## Data Availability Statement

The original contributions presented in the study are included in the article/supplementary material, further inquiries can be directed to the corresponding author/s.

## Ethics Statement

The studies involving human participants were reviewed and approved by The local ethics committee of Nagasaki University Graduate School of Biomedical Sciences. The patients/participants provided their written informed consent to participate in this study.

## Author Contributions

DM, TH, and KS conceived and designed the experiments. TM, DM, YI, KF, and WM performed the experiments. TM, MM, and TH analyzed the data. TS and JN created the experimental program. DM, TM, and TH drafted the manuscript. All authors contributed to the article and approved the finalized submitted version.

## Conflict of Interest

The authors declare that the research was conducted in the absence of any commercial or financial relationships that could be construed as a potential conflict of interest.
